# Is nosocomial infection really the major cause of death in sepsis?

**DOI:** 10.1186/s13054-014-0540-y

**Published:** 2014-10-01

**Authors:** Neil M Goldenberg, Aleksandra Leligdowicz, Arthur S Slutsky, Jan O Friedrich, Warren L Lee

**Affiliations:** Keenan Research Centre for Biomedical Science, St Michael’s Hospital, 30 Bond Street, Toronto, Ontario M5B 1 W8 Canada; Interdepartmental Division of Critical Care, University of Toronto, 30 Bond Street, Bond Wing 4-012, Toronto, Ontario M5B 1 W8 Canada; Li Ka Shing Knowledge Institute, St Michael’s Hospital, 30 Bond Street, Toronto, Ontario M5B 1 W8 Canada; Keenan Research Centre for Biomedical Science, St Michael’s Hospital, 30 Bond Street, Toronto, Ontario M5B 1 W8 Canada

## Introduction

Over 25 clinical trials for sepsis have failed [1,2], suggesting that our current understanding of its pathogenesis is incomplete. Deaths occur days to weeks after diagnosis and have been attributed to one of two phenomena [3]. First, a subset of patients succumbs to an overwhelming acute inflammatory response driven by the innate immune system, leading to death within days of the initial infection. However, most patients survive this phase and the repeated failure of anti-inflammatory therapies for sepsis (for example, anti-tumor necrosis factor antibodies [4], high-dose corticosteroids [5]) indicates that inflammation *per se* is unlikely to be a major cause of death. Most sepsis deaths occur later and have been associated with dysfunction of the innate and adaptive immune systems [6], characterized by decreased cytokine production and lymphocyte apoptosis [7]. These mechanisms have been postulated to cause immunosuppression [3,8,9], predisposing patients to fatal nosocomial infections. Based on this hypothesis, immunoadjuvant therapy to boost the immune system has been proposed recently as a therapeutic approach.

## The argument against nosocomial infection

The notion of death due to nosocomial infection is at odds with our clinical experience, in which patients with sepsis die despite broad-spectrum antibiotics and negative microbial cultures. Indeed, two studies often cited as evidence for this theory are open to alternative interpretations. The first study reported a high (~80 %) rate of infected foci in those patients dying from sepsis [10]. Yet it was unclear whether culture data reflected only postmortem or perimortem cultures, or incorporated laboratory results taken earlier during hospitalization – a period in which positive cultures would be expected. The second study did not report the incidence of positive cultures in patients who died from sepsis, a critical statistic for determining the contribution of nosocomial infection to mortality [1]. This study described three phases of mortality, divided into deaths occurring within hospital days 0 to 5 (phase I), days 6 to 15 (phase II) and days 16 to 150 (phase III). Despite the fact that phase III included the largest number of days by far, the mortality rate was highest in phase I, arguing against late nosocomial infection being the main cause of death.

A recent retrospective analysis in our own center has provided further evidence against this theory. We considered all patients admitted to the ICU who were screened for a sepsis study of heparin (Heparin Anticoagulation to Improve Outcomes in Septic Shock; ClinicalTrials.gov NCT 1648036) and subsequently died. From these patients, we selected those who actually had sepsis and looked for evidence of a secondary nosocomial infection, defined as a detected new microbial isolate prior to death. Of 26 consecutive patients dying of septic shock in a mixed medical–surgical ICU, only three (14 %) patients had evidence of a new infection at the time of death (Table 1). While our study is not definitive, taken together with other results, the theory that nosocomial infection is the predominant cause of death from sepsis seems tenuous.

**Table 1:** **Lack of evidence of nosocomial infection in a retrospective cohort of patients dying of sepsis**

**Age range (years)**	**Source of infection**	**SOFA score**	**ICU LOS (days)**	**Days from diagnosis to death**	**Days from final culture to death**	**Final culture result**	**Cause of death**	**Evidence of nosocomial infection**
70 to 79	Lung	12	16	13	1	No growth	Cancer	No nosocomial infection
60 to 69	Skin and soft tissue	9	55	55	5	No growth	CHF	No nosocomial infection
80 to 89	Genitourinary	12	1	14	2	No growth	CHF	No nosocomial infection
70 to 79	Bloodstream	15	17	17	2	Original organism	IE/sepsis	No nosocomial infection
80 to 89	Lung	18	54	73	<1	Original organism	Ischemic bowel	No nosocomial infection
40 to 49	Intra-abdominal	12	14	14	14	No growth	Liver failure	No nosocomial infection
80 to 89	Lung	11	12	10	1	No growth	MI	No nosocomial infection
70 to 79	Lung	10	5	3	1	Original organism	MI	No nosocomial infection
40 to 49	Bloodstream	21	14	4	1	No growth	MOF	No nosocomial infection
50 to 59	Bloodstream	15	16	33	3	No growth	MOF	No nosocomial infection
40 to 49	Intra-abdominal	17	4	5	1	No growth	MOF	No nosocomial infection
70 to 79	Intra-abdominal	16	42	43	3	No growth	MOF	No nosocomial infection
60 to 69	Lung	9	19	18	2	No growth	MOF	No nosocomial infection
50 to 59	Skin and soft tissue	13	10	11	1	No growth	MOF	No nosocomial infection
60 to 69	Skin and soft tissue	14	76	57	2	No growth	MOF	No nosocomial infection
<30	Skin and soft tissue	14	86	85	<1	No growth	MOF	No nosocomial infection
80 to 89	Genitourinary	16	1	1	1	Original organism	MOF	No nosocomial infection
70 to 79	Lung	4	34	22	3	No growth	Tumor lysis	No nosocomial infection
40 to 49	Intra-abdominal	7	46	74	1	New organism	MOF	Nosocomial infection
70 to 79	Intra-abdominal	5	6	6	1	New organism	MOF	Nosocomial infection
<30	Intra-abdominal	12	37	37	<1	New organism	Pancreatitis	Nosocomial infection
60 to 69	Intra-abdominal	5	1	7	3	New organism	MOF	Indeterminate
50 to 59	Skin and soft tissue	12	37	37	4	No growth	MOF	Indeterminate
40 to 49	Lung	16	68	50	6	No growth	MOF	Indeterminate
70 to 79	Lung	13	17	17	13	Original organism	MOF	Indeterminate
70 to 79	Intra-abdominal	14	3	13	<1	No growth	Sepsis	Indeterminate

The initial antibiotic therapy was checked against culture and sensitivity results to record the appropriateness of antibiotic therapy for the initial isolate. Results of the final culture before death and the time from that culture until death are indicated (<1 means the same day as death). CHF, congestive heart failure; IE, infective endocarditis; LOS, length of stay; MI, myocardial infarction; MOF, multiorgan failure; SOFA, Sequential Organ Failure Assessment.

## If not infection, what else?

### Mitochondrial dysfunction

There is substantial evidence for mitochondrial dysfunction in sepsis [11]. The theory is that if perfusion and oxygen content are adequate but organ dysfunction still exists, the cells must be unable to use oxygen. Several factors, including reactive oxygen species, hormonal deficiencies, and the impact of systemic inflammation on mitochondrial gene transcription, are thought to contribute [11,12]. Furthermore, leukocytes from septic patients have been shown to possess abnormal oxygen metabolism [13], and mitochondrial dysfunction has been associated with poor outcomes in septic shock [14]. While trials of antioxidant therapy have been unsuccessful (reviewed in [2]), further trials are needed to determine the validity of this approach.

### Microvascular leak

**Figure 1:**
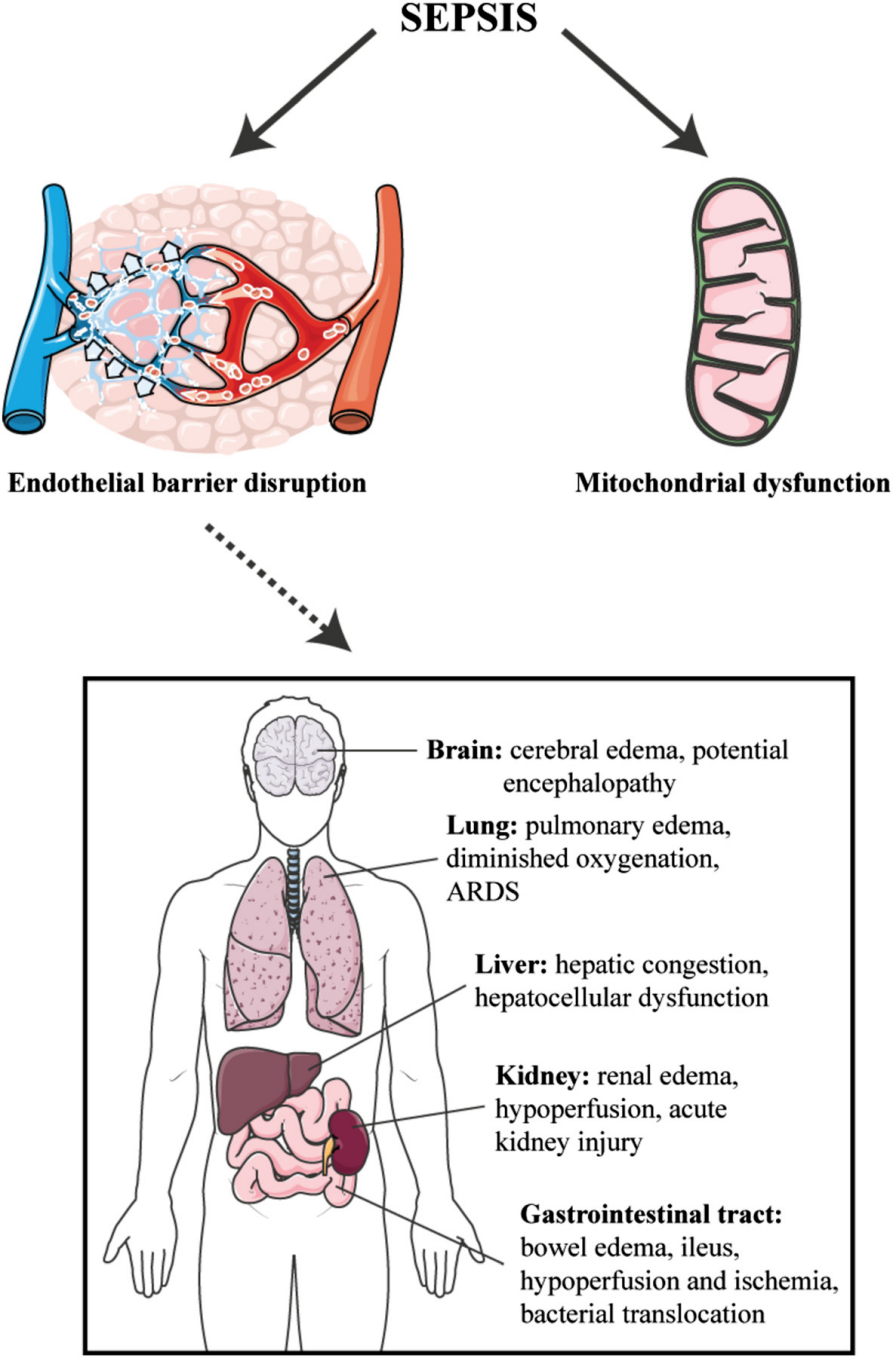
**Evidence supporting nosocomial infection as the major cause of death in sepsis is weak.** Alternative hypotheses include the role of mitochondrial dysfunction as well as systemic microvascular leak (see text). ARDS, acute respiratory distress syndrome.

Another theory for death from sepsis implicates systemic vascular leak (Figure 1) [15,16]. Loss of endothelial barrier integrity leads to tissue edema, hypoperfusion, and organ dysfunction. These features are characteristic of human sepsis but until recently were absent from animal models. Importantly, various lines of evidence for this theory exist. In mice, buttressing the endothelial barrier directly protected against death from sepsis [17,18]. In humans, limiting fluids accelerated recovery in acute respiratory distress syndrome [19], while a positive fluid balance was associated with worse outcome in sepsis [20]; most recently, in a *post hoc* subgroup of the sickest sepsis patients, albumin therapy – which would limit edema formation – was also protective [21].

## Conclusion

Sepsis has been termed a pharmaceutical ‘graveyard’ [22] due to repeated failure of human clinical trials. Despite calls for a trial of immunoadjuvant therapy, the evidence supporting nosocomial infection as the main cause of death is weak. A small study of granulocyte–macrophage colony-stimulating factor in patients with severe sepsis/septic shock observed improvements in monocyte function but no significant change in a host of clinical parameters except for the duration of mechanical ventilation [23]. Practically, if most patients who die from sepsis have sterile cultures, it is unlikely that boosting the immune system or adding additional antibiotics will improve outcomes. Further research into the contribution of nosocomial infection to sepsis mortality is thus necessary, as well as research into other potential contributors such as systemic microvascular leak.
